# Conservation of Domestic Animal Genetic Resources in China: Overview of the Status, Activities, Policies, and Challenges

**DOI:** 10.3390/life15091420

**Published:** 2025-09-10

**Authors:** Xiao Chen, Jian Lu, Wenqiang Cheng, Ming Xue, Fuqing Yu

**Affiliations:** 1 State Key Laboratory of Resource Insects, Institute of Apicultural Research, Chinese Academy of Agricultural Sciences, Beijing 100193, China; 2 National Animal Husbandry Station, Beijing 100125, China; lujian34@163.com (J.L.);

**Keywords:** domestic animal resources, diversity, conservation, governance, sustainable use and development

## Abstract

Livestock and poultry biodiversity constitutes an essential element of global biological diversity, playing a pivotal role in sustaining human livelihood and socioeconomic development. Domestic animal genetic resources in China are abundant and various. Especially, local breeds have strong adaptability to the environment and exhibit excellent traits. They are the material foundation for both the original innovation in agricultural technology and the development of modern animal husbandry. Conservation of animal genetic resources is the primary action for sustainable use and development of domestic animals. Globally, many national and international institutions have initiated a variety of conservation measures, legislation, and technical strategies. China has likewise undertaken relevant initiatives. In this paper, we summarize the current situation of domestic animal resources in China, including the current status of domestic animals, the conservation measurements, the sustainable utilization, the management policies, challenges, and suggestions for the conservation of domestic animal resources. The sustainable use and protection work on domestic animals can be incorporated with the issues of food security and sustainability, the protection of the environment and climatic change, concepts in which societal interest is continuously increasing.

## 1. Introduction

Genetic resources of domestic animals are important for world food security, considering their contribution to the livelihoods of over a billion people. General economic development and population growth and mobility in the world has increased demand for livestock products, but has also introduced pressures on the sustainability of rural environments and animal production systems [1]. A diverse resource base is critical for human survival and well-being, and a contribution to the eradication of hunger. The conservation and sustainable use of domestic animals and the fair and equitable sharing of the benefits from their use are an international concern [1,2,3].

China is one of the richest countries in domestic animal genetic resources. There were 1090 livestock and poultry breeds registered in China by 2024 (http://www.nahs.org.cn/gk/tz/202502/t20250210_452797.htm, accessed on 15 February 2025) [4]. They have remarkable performances in reproductive features, adaptability, rough feed tolerance, meat quality, etc. [4,5]. They are the basic material for animal breeding. China has also been successful in domestic animal protection. There are policies especially for domestic animal conservation. The country has established a tiered protection strategy with oversight at both the national and provincial levels to safeguard the economic, ecological, and cultural value of these resources [6]. A number of precious, rare, and endangered resources were protected with priority [4].

This review paper deals with the issue of the protection activities in China and includes an overview of the current status of domestic animals, the conservation measurements, the sustainable utilization, the management policies, challenges, and suggestions for conservation of domestic animal resources. The review is based on the national investigation of domestic animals in China.

## 2. Status of Domestic Animal Resources in China

China is home to a rich variety of domestic animal genetic resources. The National Catalog of Livestock and Poultry Genetic Resources details 17 traditional and 16 special types as domestic animals (https://zypc.nahs.org.cn/pzml/index.html, accessed on 10 October 2024). Here, the species of traditional types refer to the species raised by people in the long-term development of animal husbandry. These 17 traditional species include pigs, ordinary cattle, zebu, buffaloes, yaks, large cattle, sheep, goats, horses, donkeys, camels, rabbits, chickens, ducks, geese, pigeons, and quails. The species of special types refer to relatively new species that emerge with the market demand in recent years, or domestic animals raised in small quantities other than traditional ones. These 16 special types include sika deer, red deer, reindeer, alpacas, turkeys, guinea fowls, pheasants, partridges, Muscovy ducks, mallard, ostrich, emu, mink (non-food animal), silver fox (non-food animal), arctic fox (non-food animal), and raccoon dog (non-food animal).

**Figure 1:**
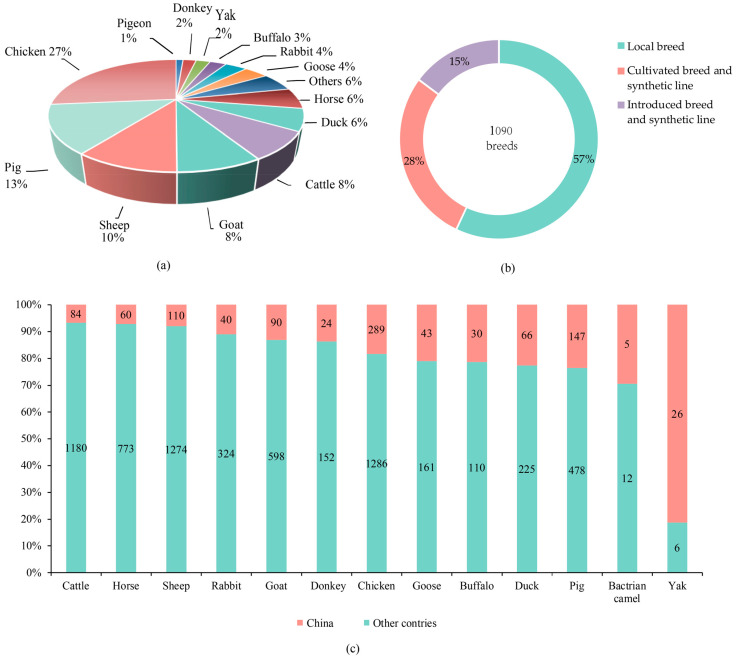
Status of domestic animal resources in China in 2024. (**a**) The proportion of the breeds of different domestic animals in China; (**b**) The proportion of the local breed, cultivated breed, crossbred and introduced breed/crossbred in China; (**c**) The proportion of the domestic animal breeds in China to the total number of breeds in the world.

China published the “Chinese Livestock and Poultry Genetic Resources Breed List” in both 2021 (2021 version, http://www.nahs.org.cn/gk/tz/202101/P020210115497616621650.pdf, accessed on 15 February 2025) and 2025 (2024 version, http://www.nahs.org.cn/gk/tz/202502/t20250210_452797.htm, accessed on 15 February 2025). According to the latest list (2024v), there are 1090 livestock and poultry breeds registered in China, including 1032 breeds of traditional species and 58 breeds of special species (Table 1 and Figure 1). The number of breeds for pigs, sheep, and chickens each exceeds 100, respectively, demonstrating rich genetic diversity. Based on their origin, breeds are categorized as local breed, cultivated breed, crossbred, introduced breed, and introduced crossbred. Among the 1090 registered breeds, there are 622 local breeds, 306 cultivated breeds and crossbreeds, and 162 introduced breeds/crossbreeds. Species in which local breeds account for over 50% include pig, cattle, yak, sheep, goat, duck, and goose [4]. The high proportion of local breeds indicates rich genetic diversity within these species in China. Species in which cultivated breeds and crossbreeds account for over 30% include pig, sheep, rabbit, chicken, quails, sika deer, red deer, pheasant, Muscovy ducks, minks (non-food animal), and raccoon dogs (non-food animal) [4]. Cultivated breeds and crossbreeds are varieties developed by breeding enterprises to meet market demands, utilizing local breeds, introduced breeds, and other resources. Cultivated breeds and crossbreeds possess high production capabilities [7].

Based on China’s Animal Husbandry Law, honeybees and silkworms also fall under the administration of the animal husbandry sector (https://www.gov.cn/xinwen/2022-10/30/content_5722639.htm, accessed on 10 October 2024). In 2025, China published the “Chinese Honeybee Genetic Resources Breed List” and “Chinese Silkworm Genetic Resources Breed List” (2024 version, http://www.nahs.org.cn/gk/tz/202502/t20250210_452797.htm, accessed on 15 February 2025). According to these lists, 39 breeds of honeybees and 307 breeds of silkworms are registered in China.

Data on Chinese domestic animal breeds have also been compared to the world’s animal genetic resources (the Domestic Animal Diversity Information System of FAO, https://www.fao.org/dad-is, accessed on 20 October 2022). As of September 2022, there are a total of 8264 existing livestock and poultry breeds in the world. In 2024, there are 147 pig breeds in China, accounting for 23.5% of the global total of 625 breeds (Figure 1) [4]. There are 26 yak breeds in China, accounting for 81.3% of the global total of 32 breeds (Figure 1). There are 90 goat breeds in China, accounting for 13.1% of the global total of 688 (Figure 1). There are 24 donkey breeds, accounting for 13.6% of the global total of 176 (Figure 1). There are 289 chicken breeds, accounting for 18.3% of the global total of 1575 (Figure 1) [4].

## 3. Conservation Activities on Domestic Animals Resources in China

### 3.1. Implemented the National Investigations on Domestic Animal Resources

**Figure 2:**
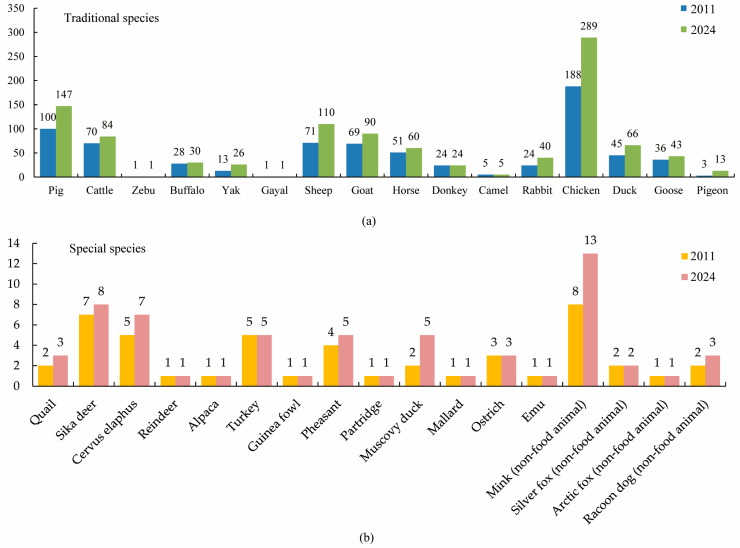
Annual change in number of different livestock species in China. (**a**) Change in number of traditional livestock species. (**b**) Change in number of special livestock species. Data source: Annals of Genetic Resources of Livestock and Poultry in China, Chinese Livestock, and Poultry Genetic Resources Breed List (2024v).

Since 1949, China has carried out three national-scale surveys on domestic animal resources. The first one was carried out in 1976–1983. Based on this survey, the “Journal of Chinese Livestock and Poultry Breeds” was published, which recorded 282 distinct breeds. The second national survey was implemented from 2006 to 2009. Based on this survey, the situation of livestock and poultry genetic resources in China at that time were recorded, and the changes in populations from 1983 to 2009 were analyzed. In 2011, the Annals of Genetic Resources of Livestock and Poultry in China was published, which recorded 747 distinct breeds [8]. From 2021 to 2023, the Chinese government implemented the third national survey. This survey aimed to find out the status, populations, and regional distribution of livestock and poultry genetic resources. Also, the characteristic and trait performance were measured and evaluated. Based on the third survey, the “Chinese Livestock and Poultry Genetic Resources Breed List (2024v)” (http://www.nahs.org.cn/gk/tz/202502/t20250210_452797.htm, accessed on 15 February 2025) and “Report on Domestic Animal Genetic Resources in China” was published [4]. According to the breed list, there are 1090 livestock and poultry breeds (Figure 2).

### 3.2. Formulating the National List of Protected Domestic Animals

**Figure 3:**
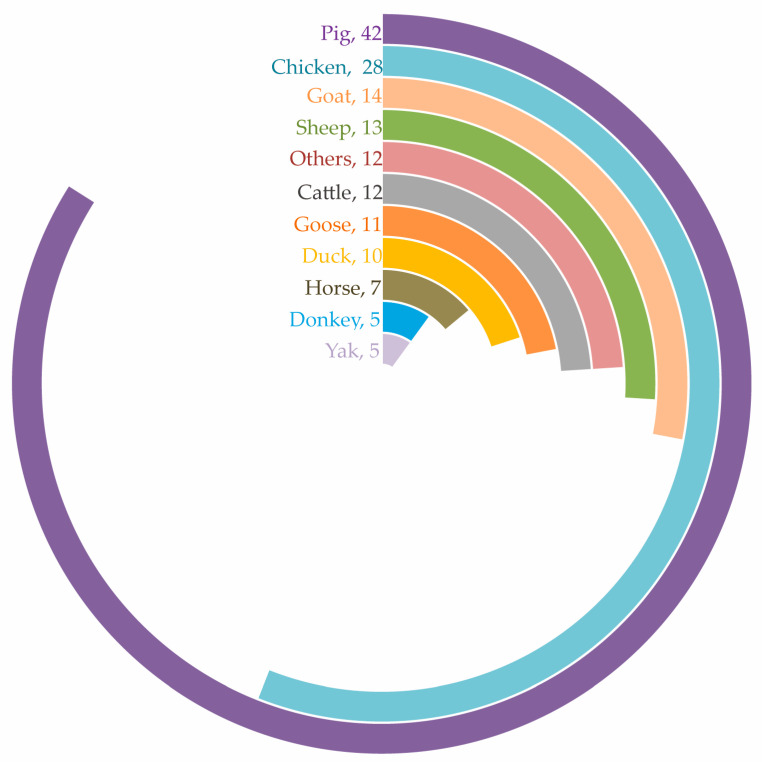
The species included in the National List of Livestock and Poultry Resource Breeds for Protection. Note: the number means how many breeds of these species are included in the list.

**Figure 4:**
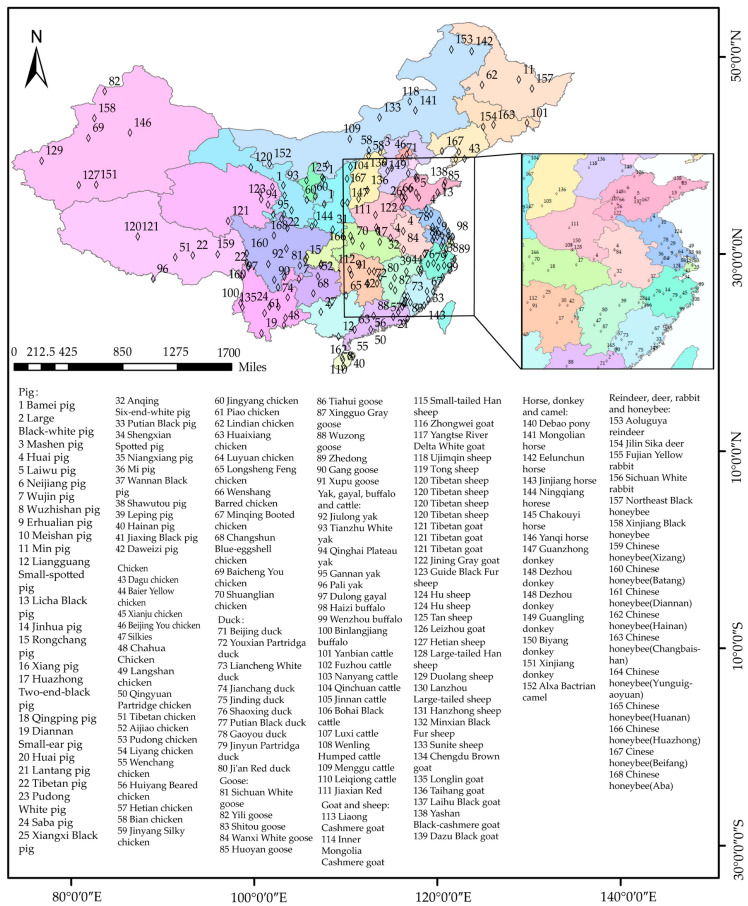
The distribution of breeds in “National List of Livestock and Poultry Resource Breeds for Protection”. The visualization was created using software ArcMap 10.8.

Since 2005, China has implemented numerous regulations for the protection and sustainable management of livestock and poultry genetic resources [6]. In China, the national and provincial governments have joined together to implement protection work for domestic animals. The national government published the “National List of Livestock and Poultry Resource Breeds for Protection”, and the provincial governments published their “Provincial List of Livestock and Poultry Resource Breeds for Protection”. Regarding criteria for inclusion in the protection list, breeds with endangered populations or those possessing outstanding traits are prioritized. Subsequently, both the national government and provincial governments have implemented protection measures for the listed breeds. Specifically, this involves establishing dedicated conservation farms for each protected breed and collecting and cryopreserving genetic materials. The government has issued technical requirements for breed protection to ensure protection effectiveness. In February 2014, the national government released the latest version of the “National List of Livestock and Poultry Resource Breeds for Protection” (https://www.moa.gov.cn/nybgb/2014/dsanq/201712/t20171219_6105903.htm, accessed on 10 October 2024), which includes 159 breeds (Figure 3 and Figure 4). At the provincial level, a total of 537 breeds were included in the “Provincial List of Livestock and Poultry Resource Breeds for Protection” [4].

### 3.3. Construction of the Protection System for Domestic Animal Resources in China

**Figure 5:**
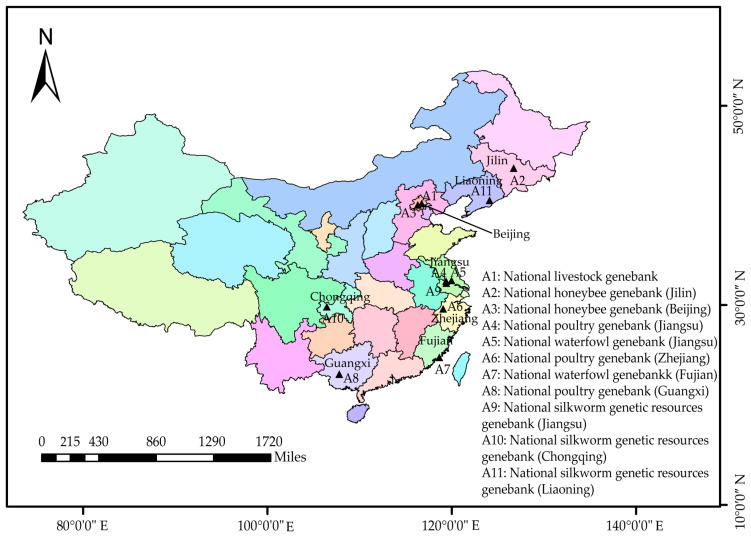
The distribution of national gene banks in China. The visualization was created using software ArcMap 10.8.

In China, the protection system includes national-level protection and provincial-level protection. The ways to protect domestic animal resources mainly include in situ conservation and cryoconservation [9]. In situ conservation, based on conservation farms, is crucial for protection and management, as it helps protect distinct and ecologically significant populations within a species [10]. Since 2024, there have been 191 national-level conservation farms and 25 national-level conservation reserves [11,12,13]. And there have been 574 provincial-level conservation farms and 52 provincial-level conservation reserves. Around 80% of the breeds on the “National List of Livestock and Poultry Resource Breeds for Protection” have been protected in the conservation farms and reserves. Also, there are 11 national-level gene banks (Figure 5) and 45 regional-level gene banks [11,12,13]. The national-level livestock gene bank conserves genetic resources for 371 breeds. The national poultry gene bank conserves genetic resources of 86 breeds. The honeybee gene bank conserves semen of 17 honeybee breeds [4].

## 4. Sustainable Use and Development of Domestic Animal Resources in China

China’s vast geographical and ecological diversity, coupled with varied animal husbandry production systems and market demands across regions, has led to distinct patterns in the utilization of livestock and poultry genetic resources [4]. In economically developed livestock regions, production primarily relies on high-yield introduced breeds (including hybrids with local breeds) and cultivated breeds [14,15,16]. Conversely, economically underdeveloped areas—particularly remote mountainous zones and unique ecological regions—depend predominantly on local breeds [17]. High-yielding-introduced breeds focus on ensuring the effective supply of livestock products, to meet the large quantity demand [16]. Local breeds focus on improving the quality level, producing special featured livestock products to meet the special flavor demand [18]. In recent years, new cultivated breed and crossbred development programs utilize both local breeds and high-yielding-introduced breeds as foundational breeding materials [19]. The resulting cultivated breed and crossbred combine rapid growth rates with premium quality attributes, representing the optimal utilization pathway for local breeds. In 2024, around 53% of local breeds have been used in cultivated breeding and crossbreeding [4].

## 5. Conservation Policies in China

In 2006, the Chinese government enacted and implemented the Animal Husbandry Law (https://www.gov.cn/xinwen/2022-10/30/content_5722639.htm, accessed on 10 October 2024). To ensure its effective implementation, China subsequently issued supporting regulations, including “Measures for the Examination and Approval of the Entry–Exit and Foreign Cooperative Research and Utilization of Domestic Animal Genetic Resources” (https://www.gov.cn/zhengce/zhengceku/2008-09/04/content_2880.htm, accessed on 10 October 2024), “Measures for the Administration of Conservation Farms, Protection Zones, and Gene Banks of Domestic Animal Genetic Resources” (https://www.moa.gov.cn/gk/nyncbgzk/gzk/202210/P020221010466236429731.pdf, accessed on 10 October 2024), and “Procedures for the Examination of New Livestock and Poultry Varieties and the Identification of Domestic Animal Genetic Resources” (https://www.moa.gov.cn/gk/nyncbgzk/gzk/202210/P020221012408660197626.pdf, accessed on 10 October 2024).

The Animal Husbandry Law underwent amendments in 2015 and 2022. It establishes comprehensive provisions for the protection and management of domestic animal genetic resources. The law mandates a national conservation system for domestic animal genetic resources and legally defines conservation responsibilities. Aligning with strategies for seed industry development, and drawing on international conventions, the Chinese government enacted the following laws, including the “Biosafety Law of the People’s Republic of China” (https://wb.flk.npc.gov.cn/flfg/PDF/497947320c8547be92ef45d0893ae8f1.pdf, accessed on 10 October 2024), and the “Rural Revitalization Promotion Law of the People’s Republic of China” (http://www.feixian.gov.cn/__local/F/7F/48/E935960CA8D843E67EB52530F3D_F8EBF9C6_3AC4D.pdf, accessed on 10 October 2024). These laws impose strict regulations on the management of biological genetic resources (including livestock and poultry), covering biosafety, national sovereignty, resource protection, and security review mechanisms. Currently, China’s legal framework for domestic animal genetic resources comprises “The Animal Husbandry Law” and its supporting regulations, departmental regulatory documents, and sequentially enacted laws and regulations related to domestic animal genetic resource management.

## 6. Challenges in Sustainable Use and Development of Domestic Animals in China

### 6.1. The Utilization of Local Breeds

The current utilization of local breeds is not sufficient. Around half of the local breeds have not been used in cultivated breeding work. Many Chinese local pig breeds and chicken breeds are valued by consumers for their favorable meat quality, remarkable performance in reproductivity, and adaptation to harsh conditions [20,21]. However, compared to commercial breeds, local breeds have disadvantages, such as low feed conversion efficiency and slow growth rates [20,21]. This results in lower economic benefits for local breeds than for commercial breeds. Therefore, many local breeds are not being transformed into industrial development. The identification of excellent characteristics of local breeds is not enough and the genes behind them are barely known, which makes it difficult to use them in breeding programs. Local breeds are farm animal populations that have adapted to local conditions, including traditional agricultural production systems and environments [22]. They originate from certain geographical regions, are adapted to these regions’ environmental conditions, and are often utilized therein [1]. During the past few decades, the population of local breeds has decreased due to the requirements of intensive livestock farming and global economic development [23]. It is estimated that nearly 18% of local breeds in China are in an endangered state [4]. Local breeds are frequently valued as genetic resources for future animal breeding and economic importance, but they also have cultural value [24]. However, currently, the excellent traits of local breeds have not been transformed into industrial development. The best way to conserve local domestic animals is to utilize them effectively. There is still long way to transfer this concept to practice.

### 6.2. The Genetic Mechanism of Excellent Traits

The studies on clarifying the genetic mechanism of excellent traits are insufficient. Although habitat preservation and restoration are the primary means of conserving biodiversity, the advancement of molecular biological technologies offer a variety of novel tools for identifying biodiversity hotspots; thus, they support conservation efforts [21,25,26,27,28,29,30,31]. The approaches of conservation genetics and genomics have been widely used in the protection of threatened species and provide effective conservation suggestions [30,32,33,34,35,36,37,38]. China has insufficient investment in the basic theory of livestock and poultry conservation genetics and genomics, discipline construction, and other fields, and the cryopreservation of semen of pig, horse, donkey, and other embryos and poultry chimeras is still immature, which needs to be tackled urgently.

### 6.3. The Resource Disappearance Risk

The risk of resource disappearance increased due to overdevelopment, ecological degradation, and climate change. Economics may not be the only explanation for the loss of local breeds. International agencies may be overly enthusiastic about modem livestock methods to the detriment of traditional indigenous species [24,29,39,40]. The governments which introduced cultivated breeds may overestimate the benefits of moving to more modern livestock methods [24,29,39,40]. Government support of intensive livestock farming may have intentionally or unintentionally contributed to the loss of local breeds [24,39]. Anyhow, with the rapid advancement of urbanization, environmental changes, introduction of foreign breeds, and other factors, the population of domestic animals have decreased significantly [41]. The populations of around half of the local breeds show a decrease trend in China in the past 10 years [4]. Around 18% of the local breeds are in an endangered state [4].

### 6.4. The Biosafety Risks

The biosafety risks are serious. Biosafety risks have diverse sources, including microbial contamination, unintended consequences of gene-editing technologies, threats posed by invasive alien species, and illegal trade of germplasm resources. These risks may not only cause irreversible damage to local ecosystems but also jeopardize human health and agricultural production security, ultimately undermining socioeconomic stability and development [42]. The introduction of livestock and poultry from abroad may carry pathogens not yet present in the host country, such as African swine fever and highly pathogenic avian influenza [43]. If these diseases enter due to quarantine loopholes, they could trigger large-scale epidemics, leading to increased mortality rates in local livestock, production stagnation, and even threats to human health [43]. For example, the 2018 cross-border transmission of African swine fever in China caused an outbreak resulting in economic losses exceeding hundreds of billions [43].

## 7. Suggestions for Sustainable Use and Development of Domestic Animals

### 7.1. Establish Regular Investigations on Domestic Animal Resources

The domestic animal genetic resources are variable and renewable resources. With the changes in climate, environment, consumption demand, and other factors, the breeding direction would constantly changing [44]. Thus, it is necessary to establish a regular investigations mechanism, collect genetic materials regularly, and assess the impact of introduced breeds on the local breeds [22]. In this way, the government would be aware of the domestic animal genetic resource changes in real time and implement scientific management in time.

### 7.2. Develop Programs for the Conservation of Domestic Animals Resources

Firstly, the government should conduct regular evaluations of the endangered status of livestock populations. Upon identifying any breed at risk, immediate conservation efforts must be initiated to prevent extinction. Secondly, it is necessary to develop programs on the in situ conservation of domestic animal genetic resources. Effective in situ conservation should keep all the characteristics of breeds in the original state to ensure the breeds are not lost [9]. The third is to develop studies on cryoconservation of domestic animals. The technologies of frozen semen, frozen embryos, somatic cloning, and embryonic stem cells should be paid more attention. Fourthly, studies should be developed to explore the characteristics of domestic animals. It is suggested to focus on traits such as high reproduction rate, good meat quality, and strong adaptability, and cultivate new breeds with unique characteristics and outstanding production performance [45,46]. The fifth is to scientifically assess the impact of introduced breeds on the local breeds. In 2025, Qiu et al. elucidate the genomic bases of bidirectional introgression in pigs resulting from the intra-continental trade between China and Europe. The results showed the widespread dispersal of Chinese haplotypes and SVs across European pig breeds, and the potential phenotypic effect of European haplotypes and SVs on commercial traits in Chinese breeds [47]. For the large-scale introduction of foreign breeds, it is necessary to strengthen the disease detection, environmental supervision, etc., and carry out a scientific and systematic assessment to ensure the biological safety of local breeds [48].

### 7.3. Clarify the Responsibility for Conservation of Domestic Animal Resources

Clarifying the responsibility would promote the conservation of domestic animal resources. The central government and the local government are responsible for the implementation of support measures. It is recommended that the government provide different financial support according to the different livestock breeds [1]. In this way, it would ensure more breeds were protected with a certain amount of funds. The ideal situation is that all the breeds on the protection list are protected. Moreover, a specific protection plan should be made for each breed.

### 7.4. Promote the Development and Utilization of Domestic Animal Genetic Resources

With the development of molecular biology techniques, more accurate identification work of livestock and poultry breeds would be carried out, including phenotypic traits and genomic information [45,49,50]. It is recommended to establish a DNA (deoxyribonucleic acid) database of livestock and poultry genetic resources. It is also suggested to explore the formation mechanisms of excellent traits. The government, scientists, and breeders would utilize modern molecular biology techniques to promote the utilization of livestock and poultry resources. Furthermore, livestock and poultry resources could be combined with traditional cultures to promote the development of local livestock and poultry resources and boost the local economy. It suggested to build an information service platform for livestock genetic resources to facilitate communication and sharing among different databases.

### 7.5. Cultivate Professionals for Sustainable Use and Develop of Domestic Animal Resources

It is necessary to persist in cultivating technicians for the protection and development of livestock and poultry genetic resources over the long term. Various policies and incentive mechanisms should be established so that technicians are willing to work in this field permanently. At the same time, the government should establish corresponding policies to support universities and research institutes in setting up majors related to the protection and utilization of livestock and poultry genetic resources, and continuously promote the development and innovation of these majors [1,51]. On the other hand, companies should be encouraged to carry out the work of protecting livestock and poultry resources and breeding. The government should build a development platform and support companies in terms of policies, projects, funds, and other aspects.

### 7.6. Awareness of Domestic Animal Keepers for Maintaining Pure Germplasm

Pure germplasm serves as the genetic foundation for breeding new livestock and poultry varieties with enhanced disease resistance and superior production performance [52,53]. To preserve desirable qualities, unique flavors, and specific traits, some pure germplasm must be utilized directly through purebred selection. To encourage purebred conservation, the government should establish corresponding economic incentives, e.g., direct subsidies, tax reductions. On the market side, it is better to implement a premium pricing system for quality products. To enhance directly the utilization of pure breeds, livestock and poultry breeding enterprises are encouraged to produce high-value goods using purebreds to meet market demand. Also, the livestock and poultry breeding enterprises are encouraged to breed specialized strains with outstanding traits (e.g., disease resistance, high fertility, product excellence) for commercial crossbreeding or new breed/line development. Direct utilization of pure breeds not only preserves domestic animal genetic resources but also elevates breed quality, creating a virtuous cycle of conservation-through-utilization and development-through-use.

## 8. Conclusions

Domestic animal genetic resources, as the common heritage of humankind, constitute a vital component of global biodiversity and represent strategic germplasm resources for modern agriculture and animal husbandry. This paper comprehensively reviews the status of China’s domestic animal genetic resources, including the current status, the conservation measurements, the sustainable utilization, the management policies, challenges and suggestions for future conservation strategies. The protection work on domestic animals can be incorporated with the issues of food security and sustainability, the protection of the environment and climatic change, concepts in which societal interest is continuously increasing.

## Figures and Tables

**Table 1:** Number of breeds of domestic animals in China in 2024.

Classification	Species	Local Breed	Cultivated Breed	Crossbred	Introduced Breed	Introduced Crossbred	Total
Traditional domestic animals	Pig	89	32	18	6	2	147
	Cattle	58	11	0	15	0	84
	Zebu	0	0	0	1	0	1
	Buffalo	27	0	0	3	0	30
	Yak	24	2	0	0	0	26
	Gayal	1	0	0	0	0	1
	Sheep	58	38	0	14	0	110
	Goat	69	15	0	6	0	90
	Horse	29	14	0	17	0	60
	Donkey	24	0	0	0	0	24
	Camel	5	0	0	0	0	5
	Rabbit	8	13	5	10	4	40
	Chicken	140	5	104	8	32	289
	Duck	42	0	16	1	7	66
	Goose	32	1	4	0	6	43
	Pigeon	6	0	3	3	1	13
	Quail	0	0	1	2	0	3
Special domestic animals	Sika deer	1	7	0	0	0	8
	Red deer	3	3	0	1	0	7
	Reindeer	1	0	0	0	0	1
	Alpaca	0	0	0	1	0	1
	Turkey	1	0	0	2	2	5
	Guinea fowl	0	0	0	1	0	1
	Pheasant	2	2	0	1	0	5
	Partridge	0	0	0	1	0	1
	Muscovy duck	1	0	2	1	1	5
	Mallard	0	0	0	1	0	1
	Ostrich	0	0	0	3	0	3
	Emu	0	0	0	1	0	1
	Mink (non-food animal)	0	8	0	5	0	13
	Silver fox (non-food animal)	0	0	0	2	0	2
	Arctic fox (non-food animal)	0	0	0	1	0	1
	Racoon dog (non-food animal)	1	2	0	0	0	3
Total		622	153	153	107	55	1090

## Data Availability

Not applicable.
